# Past five-year trend, current prevalence and household knowledge, attitude and practice of malaria in Abeshge, south-central Ethiopia

**DOI:** 10.1186/s12936-015-0749-5

**Published:** 2015-06-03

**Authors:** Fisseha Yimer, Abebe Animut, Berhanu Erko, Hassen Mamo

**Affiliations:** Department of Microbial, Cellular and Molecular Biology, College of Natural Sciences, Addis Ababa University, PO Box 1176, Addis Ababa, Ethiopia; Aklilu Lemma Institute of Pathobiology, Addis Ababa University, PO Box 1176, Addis Ababa, Ethiopia

**Keywords:** Attitude, Control, Ethiopia, Knowledge, Malaria, Practice, Prevalence, Walga Health Centre

## Abstract

**Background:**

In Ethiopia malaria remains a leading cause of outpatient consultation despite massive control efforts. This study was aimed at analysing 5-year retrospective trend and current prevalence of malaria as well as community knowledge, attitude and practice (KAP) in Walga Health Centre (WHC) catchment area in Abeshge District, south-central Ethiopia.

**Methods:**

A cross-sectional, household survey was conducted to determine malaria prevalence and KAP in December 2013. Further, malaria cases reported from WHC in 2008–2012 were extracted. A multi-stage, random sampling technique was used to select study participants from four *kebeles.* Of 800 participants, 400 were interviewed to assess their KAP about malaria and the other half were recruited for malaria microscopy.

**Results:**

Overall, 11,523 (33.8 %) slide-confirmed malaria cases were reported (no fatalities) among 34,060 outpatients diagnosed in 2008–2012. There was successively significant decline in malaria prevalence from 2009 onwards although a significant rise was noticed in 2009 compared to 2008 (*p* <0.0001). Male malaria suspects (17,626) were significantly higher than of females (16,434) (*p* = 0.0127) but malaria prevalence was not significantly variable between sexes. Individuals who were ≥15 years constituted 44.9 % of the patients. Although most participants (78.8 %) associated mosquito bites with malaria, the remaining mentioned exposure to rain or body contact with malaria patients as causes of malaria. Mosquito nets, draining stagnant water and indoor residual spraying were the most frequently mentioned malaria preventive measures. In the parasitological survey, a single individual (0.25 %) with mixed *Plasmodium falciparum-Plasmodium vivax* infections was found.

**Conclusion:**

Although malaria remains a primary cause of outpatient admission in WHC, the retrospective data showed a significantly declining trend. This together with the very low prevalence in the current parasitological survey suggests the effectiveness of ongoing control interventions in the locality.

## Background

The global burden of malaria is substantially declined, but in Africa the disease remains a major public health problem [1]. In Ethiopia, although great progress has been achieved in recent years due to scale-up of control interventions, malaria is still a formidable health challenge in the country. In 2012, countrywide, 1,822,581 confirmed cases and 253 deaths were attributed to malaria [2]. Surprisingly, in areas below 2000 m above sea level (masl) an increase in malaria prevalence was observed in 2011 (1.3 %) compared to that in 2007 of 0.9 % [3]. *Plasmodium falciparum* and *Plasmodium vivax*, which are distributed all over the endemic regions of the country, account for about 60 and 40 % of malaria cases, respectively [4].

Insecticide-treated bed nets (ITNs) are among major malaria control interventions globally. Reports show that ITNs, especially long-lasting insecticidal nets (LLINs), reduce the incidence of clinical malaria by 50 % [5, 6]. Indoor residual spraying (IRS) is vital in malaria control next to ITNs. Evidence confirmed that malaria control using IRS has made epidemics less frequent and resulted in overall malaria transmission reduction or even eradication (reviewed by Mobaso et al. [7]). IRS is effective for 3 to 6 or 9 to 12 months in some cases, depending on the insecticides used and the type of surface on which it is sprayed [8]. In 2011, 153 million people were protected by IRS around the world [9]. In Africa, countries such as South Africa have achieved a notable decline in the level of malaria transmission in some provinces through elimination of the major local malaria vectors using IRS [10].

In some settings there is combined use of LLINs and IRS in an effort to further accelerate malaria reduction. This is based on findings of limited number of observational studies that reported added protection conferred to those who received both interventions relative to those who received only one although other studies showed no such effect [11]. Whether there was better protection by the combined use of LLIN and IRS or not these studies were not randomized trials and there was no control for potential confounding factors and bias. Later, a number of randomized studies have been conducted in different sub-Saharan African countries to determine the added efficacy of the two tools and found diverging results calling for further investigation [12].

Besides their clinical efficacy the use and effectiveness of ITNs and IRS, singly or in combination, may be influenced by a range of environmental, technical, social, and behavioural factors [13, 14]. Net washing and wall replastering after IRS determine insecticide longevity and, if done at a higher frequency before the recommended time, can greatly reduce the effectiveness of ITNs and IRS, respectively [15]. There have been a considerable number of reports about knowledge, attitude and practice (KAP) related to malaria and its control from different parts of Africa. These reports [16–18] concluded that misconceptions concerning malaria still exist among communities, particularly in rural areas and that practices for the control of the disease have been unsatisfactory. The flow of malaria cases to health facilities is likely to be affected by patients’ treatment-seeking behaviour as well as the depth and quality of malaria health education provided to the community. Attitudes towards malaria interventions, structural factors affecting delivery and uptake, and other related factors govern the success of malaria control efforts. These factors have been studied using quantitative as well as qualitative approaches [19, 20]. Social and cultural factors that shape malaria treatment-seeking behaviour in sub-Saharan Africa are comprehensively reviewed [21, 22].

It is, therefore, emphasized that the value of adequate knowledge of malaria is vital to ensure that people apply preventive measures, and seek prompt and appropriate treatment for themselves and their family [23, 24]. The understanding of the possible causes, modes of transmission, and individuals’ decisions about adoption of preventive and control measures vary from community to community and among individual households (HHs) [25–27].

Reliable data on past and up-to-date malaria burden coupled with community KAP help evaluate the implementation and effectiveness of proven control interventions of the disease in a locality. Moreover, understanding community KAP and past malaria trend in a given locality is vital to issue early warnings concerning outbreaks as well as scale-up of control interventions to sustainably contain and eliminate the disease. Little work has been done to this end in Abeshge, south-central Ethiopia. The present study was, therefore, aimed at assessing the past 5-year (2008–2012) picture and contemporary asymptomatic carriage of malaria, together with community KAP in Abeshge.

## Methods

### Study design

The study was designed to analyse 5-year (2008–2012) malaria trend at Walga Health Centre (WHC) and collect current information (KAP and malaria slides). There were three data sources.

### Study area and population

The cross-sectional study was conducted in four rural *kebeles* (lowest administrative units) in Abeshge District, south-central Ethiopia. The district has generally a midland climate with an altitudinal rage of 1100–2300 masl, although there are some lowland areas (1100–1500 masl). It covers an area of 61,016 ha of which the mid- and lowlands constitute roughly 85 and 15 %, respectively [28]. The total population of the district, which constitutes two urban and 26 rural *kebeles*, is 72,917 of which 37,187 (51 %) and 35,730 (49 %) were females and males, respectively [29]. Its mean annual temperature is 23.2 °C, with mean annual minimum and maximum temperatures of 18.0 and 28.3 °C, respectively. The mean annual rainfall is 801–1400 mm [30]. Maize, *tef* (*Eragrostis teff*) and pepper are produced for food and income. A small-scale animal husbandry is also practiced.

The cross-sectional study was conducted in the catchment area of WHC which is about 27 km from Wolkite Town, the capital of Gurage Zone. Four rural *kebeles* (Walga, Borer, Jeju, and Nacha Qulit) with a total population of 13,861 in 2880 HHs were involved in the study. Each village has, on average, 720 HHs and 3465 people. Male and female inhabitants were about 6792 (49 %) and 7069 (51 %), respectively [29]. The area lies at a latitude and longitude of 08°19′N 37°28′ E and 07°56′N 37°37′ E, respectively, at 1500–1800 masl.

### Sample size

The minimum sample size, 384, was estimated using the formula: $n={Z}^2\times \frac{P\left(1-P\right)}{m^2}$ [31] on the assumption that 50 % of the respondents have knowledge about the cause and transmission of malaria; and malaria prevalence estimate of 50 % (*p* = 0.5) at 95 % confidence interval (CI) Z = 1.96) and 5 % margin of error (m = 0.05). With a response rate of 96 %, the estimated sample size was 400 (384/0.96), and this was rounded up to a maximum sample size of 800 (400 individuals for parasitological survey and 400 HH heads or responsible adults for KAP) within randomly selected 400 HHs. To be more clear two individuals were sampled per HH, one for the KAP (HH head) and another for malaria detection. For the latter, any individual aged one year or above was randomly sampled through a ‘box-draw’ method. The sample design adopted for the survey was a multi-stage, cluster, random sampling technique with the health centre with its catchment *kebeles* as the first-stage unit, villages (sub-*kebeles*) as the second-stage unit and HHs as the third-stage units were selected to determine the representative sample size.

From the four eligible rural *kebeles* with 2880 HHs in the catchment of WHC, 20 villages, each with 60 HHs, were selected randomly*;* a total of five clusters (villages) from each *kebele*. The survey was aimed at reaching 20 HHs in each cluster. The frame used for this purpose was the list of villages prepared by the respective *kebele*’s administration offices. A HH list for the entire village was compiled and this list was used to randomly assign HHs for data collection process. The shared HHs for each village were divided by the total number of HHs in a given village to determine a sampling interval for selecting HHs. Accordingly, every third HH in each cluster was selected using systematic random sampling technique. An individual eligible for the parasitological survey was a member of a HH, ≥1 year(s) old, who volunteered to give blood films for malaria diagnosis. At each HH, the head (male or female) was interviewed. In the event that the head of a HH was away, an adult (18 years or older) was selected for the interview. People who stayed for at least six months in the selected *kebeles* with no history of anti-malarial drug treatment within the previous two weeks were included in the study. Relatives/guests who joined HH members during the study period, mentally sick people and children younger than 18 years were excluded from the KAP study.

### Data collection

#### Retrospective health facility data

In WHC peripheral blood is routinely examined for malaria parasite detection according to the standard operating procedure of the country. Data on the past 5-year malaria trend were captured from the heath centre for this study.

#### KAP

A structured pre-tested questionnaire containing both close- and open-ended questions was developed from earlier studies related to malaria [32] and administered to 400 randomly selected HH heads in December 2013 to collect information regarding sociodemographics and KAP of HHs pertaining to malaria control. Data were checked for completeness, and incomplete questionnaires were returned to data collectors for correction by revisiting the concerned HHs. Five per cent of the interviewed HHs were randomly selected and re-interviewed to further assure data quality.

#### Malaria slides

An experienced laboratory technician was recruited to collect finger-prick blood samples. Thin and thick blood smears were prepared, Giemsa-stained and examined for malaria parasites using Olympus light microscope (GT Vision Ltd, Japan) as per established protocol [33]. A slide was regarded as negative after 100 fields had been examined without finding parasites and *Plasmodium* species was determined for a positive slide. For quality control, a second reading of 5 % randomly selected slides was done by an experienced malaria microscopist who was blinded to the diagnosis of the first slide reader.

### Data entry and analysis

Data were checked for completeness and consistency, and entered (twice) into statistical program for social sciences version 20.0 for Windows (SPSS Inc, Chicago, IL, USA). The Chi-squared (*X*^*2*^) test was used to test differences in retrospective malaria prevalence between years, seasons, sexes, and age groups.

### Ethical considerations

The study was approved by the College of Natural Sciences Institutional Ethics Review Board, Addis Ababa University. Informed consent was obtained from adult study participants. Able children gave their assents and parents consented for them. Blood specimens were collected by trained staff and the single malaria slide-positive participant was treated free of charge as per the national guideline [34].

## Results

### Retrospective data

Among 34,060 patients diagnosed for malaria (between February 2008 and December 2012) 11,523 (33.8 %) were slide-positive. On average 6812 febrile and 2305 malaria-confirmed cases visited the health facility each year. The average monthly malaria prevalence was 2.8 %. However, the number of suspected and confirmed cases showed a fluctuating trend in the years (Table 1). Compared to the 2008, there was a significant rise in the number of suspected and confirmed malaria cases in 2009 (*X*^2^ = 17.596, *p* <0.0001). Similarly, significantly higher number of febrile patients visited the health facility in 2010 than in 2009 (*X*^2^ = 6.388, *p* = 0.0115) although the reverse was noted in terms of malaria prevalence. There was a significant decline in the number of suspected and confirmed cases of malaria in 2012 compared to the previous years (*p* <0.0001). There was successive reduction in malaria prevalence from 2009 onwards.

**Table 1:** Suspected and slide-confirmed annual malaria cases at Walga Health Center, Abeshge District, South-Central Ethiopia, from 2008–2012

Year	Number of examined	Slide-positive^a^			Infection category		
		Male (*N* = 17626)	Female (*N* = 16434)	Total	*P. falciparum* mono	*P. vivax* mono	Mixed
	no (%)	no (%)	no (%)	no (%)	no (%)	no (%)	no (%)
2008	6504 (19.5)	1360 (51.3)	1293 (48.7)	2653 (40.8)	916 (34.5)	1687 (63.6)	50 (1.9)
2009	7167 (21.4)	1715 (51.5)	1618 (48.5)	3333 (46.5)	1634 (49.0)	1646 (49.4)	53 (1.6)
2010	8864 (26.5)	1996 (52.1)	1838 (47.9)	3834 (43.3)	2531 (66.0)	1265 (33.0)	38 (1.0)
2011	5816 (17.4)	547 (56.3)	425 (43.7)	972 (16.7)	373 (38.4)	593 (61.0)	6 (0.6)
2012	5709 (16.8)	405 (55.4)	326 (44.6)	731 (12.8)	435 (59.5)	293 (40.1)	3 (0.41)
Total†	34,060 (100)	6023 (52.3, 34.1)	5500 (47.7, 33.5)	11,523 (33.8)	5889 (51.1)	5484 (47.6)	150 (1.3)

*no* number of individuals, % percentage, *N* total males/females examined in the 5 years

^a^in the health centre record the number of total males or females examined in each year was not sorted out by sex, only slide-positives were available. So the proportion indicated here is out of total, it is not for the proportion of infected individuals in each sex category. † the first numbers in the parentheses (columns 3 & 4) refer to the proportion of males/females from the total slide-positives in each group. The second number refers to the total slide-positives among males/females from the totals examined in each group

In terms of sex, 17,626 (51.7 %) of the total patients examined were males and 16,434 (48.3 %) were females. Of the total malaria slide-positives, 52.3 % (6023/11,523) were males and 47.7 % (5500/11,523) females. The data (Table 1) shows that overall, more males were examined than females and consequently more males were found infected. The difference was statistically significant (*X*^2^ = 15.738, *p* <0.0001). However, although malaria prevalence is slightly higher among males (34.2 % (6023/17,626) than females (33.5 % (5500/16,434) the variation was not statistically significant. For each year, the number of malaria-positive males was higher than that of females although statistical significance was noticed only for years 2010 (*X*^2^ = 23.755, *p* <0.0001), 2011 (*X*^2^ = 9.976, *p* = 0.0016) and 2012 (*X*^2^ = 5.485, *p* = 0.0192). In the health centre’s record, the number of total males or females examined in each year was not sorted by sex, only slide-positives were available. As a result, it was not possible to calculate the annual malaria prevalence in each sex category.

Malaria was detected in all age groups, but 44.9 % of the patients belonged to the age group over 14 years, followed by the under-five (38.3 %) and five to 14 years (16.8 %). These differences between the age groups were statistically significant (*p* <0.0001). However, the total number of examined patients in each age group could not be obtained and the proportion of slide-positive cases among each group (prevalence) could not be determined. The local health record system showed only the number of malaria cases per age group.

**Fig. 1:**
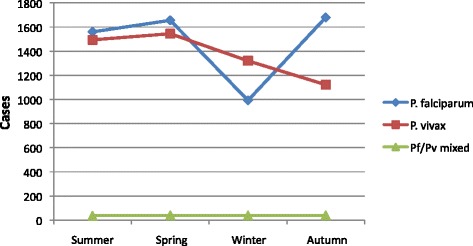
Seasonal profile of *P. falciparum, P. vivax* and mixed infections at Walga Health Centre, Abeshge; South-Central Ethiopia, from 2008–2012

The number of malaria-suspected and -infected cases varied with season (Fig. 1). The highest number of cases was registered in mid-September through November shortly after the heavy rainy season. Overall, 28.1 % of the cases occurred in this season. On the other hand, the lowest number of malaria cases (20.0 %) was observed in the driest season (December–February). During the heavy (June–August) and small (March–May) rainy seasons the number of slide-positives was 26.8 and 24.7 % respectively. The interseasonal variations were significant (*p* <0.0001) except when June–August and September–November data were compared. However, the actual number of patients examined per season was not available in the health centre making it difficult to calculate the seasonal malaria prevalence. Consequently it was not possible to ascertain the real seasonality of malaria in the study area. But it is clear that a considerable number of confirmed malaria cases were observed in all reasons.

On the whole, 51.1 and 47.6 % of the malaria cases were attributed to *P. falciparum* and *P. vivax* mono-infections, respectively. The difference was statistically significant (*p* = 0.002). Mixed *P. falciparum* and *P. vivax* infections were very low (1.5 %). A relative seasonal/annual dominance of *P. falciparum* or *P. vivax* was noticed depending on the season/year in question (Tables 1 and Fig. 1). While the frequency of *P. vivax* mono-infections in 2008 (63.6 %) and 2011 (61.0) was significantly higher than that of *P. falciparum* (34.5, 38.4 % respectively), the reverse was observed in 2010 and 2012 (*p* <0.0001 in each case). Whereas in 2010 the proportion of *P. falciparum* and *P. vivax* mono-infections was 66.0 and 33.0 %, in 2012 it was 59.5 and 40.0 %, respectively. *P. vivax* was slightly more prevalent in 2009 but the difference was not statistically significant (*p* = 0.8805).

About 26.4, 28.1 and 28.5 % of the *P. falciparum* cases were recorded in June–August, September–November and March–May, respectively. Significantly lower *P. falciparum* cases (16.8 %) were detected in the dry season compared to all other seasons (*p* <0.0001 in all cases). Except for the dry season, *P. falciparum* infections lacked a distinct seasonal pattern. Similarly, for *P. vivax* the highest cases were in September–November followed by June–August; both were significantly higher than that in both dry season and immediately after. The *p*-value for the ‘heavy-rains’ and dry season was 0.003, for the rest it was <0.0001. But, unlike *P. falciparum* the frequency of *P. vivax* was not the lowest for the dry season although it was comparably lower (24.1 %) than that of the foregoing rainy/wet seasons. Except for the dry season, *P. falciparum* was the dominant species, although the difference was statistically significant only for March–May (*X*^2^ = 74.346, *p* <0.0001). In the dry season, *P. vivax* cases (56.1 %) were significantly higher than that of *P. falciparum* (*X*^*2*^ = 30.979, *p* <0.0001).

### Malaria prevalence in the household survey

A total of 400 blood films were obtained from consenting afebrile individuals residing in the visited 400 HHs (Table 2). Two-hundred and thirty-one (57.7 %) blood donors were females. Most (46.3 %) of the blood film providers were in the age range over 14 years, while the least (10.8 %) were under-five. The overall prevalence of malaria was 0.25 %, which was contributed by only one person (male, 18 years old) asymptomatically infected with mixed *P. falciparum* and *P. vivax*.

**Table 2:** Socio-demographic characteristics of inhabitants (*n* = 400) diagnosed for malaria in Walga Health Center catchment area, Abeshge District, South-Central Ethiopia, 2013

Socio-demographics	No of examined (%)	No of slide positive (%)	*P. falciparum* mono	*P. vivax* mono	*Pf-Pv mixed*
Sex					
Male	169 (42.3)	1 (0.25)	–	–	1
Female	231 (57.7)	0 (0.0)	–	–	–
Total	400 (100)	1 (0.25)	–	–	1
Age (year)					
<5	43 (10.7)	0 (0.0)	–	–	–
5–14	47 (11.8)	0 (0.0)	–	–	–
≥15	310 (77.5)	1 (0.25)	–	–	1
Total	400 (100)	1 (0.0)	–	–	1

### KAP of households

In the cross-sectional KAP survey, a total of 400 HH heads/their delegates were interviewed out of which seven HHs were excluded from analysis because of incompleteness. Thus, the completed questionnaires were from 393 HHs resulting in a response rate of 98.25 % (Table 3). Two-hundred twenty one respondents (56.2 %) were females. More than half (51.4 %) of the respondents were in the age category 18–25 years followed by the 26.5 % who belonged to the 26–35 years age group. HH size ranged from 1 to 6 persons with a mean of 4.4 per HH (median 5 and mode 6). About 43.5 % of the participants were farmers, 31.6 % primary school complete and 26.7 % illiterate. Regarding housing, 104 (26.5 %) lived in houses with thatch roof and 289 (73.5 %) in houses with corrugated iron sheet. Three-hundred and eighty-one (96.9 %) houses were made of walls with wood and mud.

**Table 3:** Socio-demographic characteristics of inhabitants (*n* = 393) interviewed for malaria KAP in Walga Health Center catchment area, Abeshge District, South-Central Ethiopia, 2013

Socio-demographics	Frequency (%)
Sex	
Male	172 (43.8)
Female	221 (56.2)
Age	
18–25	202 (51.4)
26–35	104 (26.5)
≥36 years	87 (22.1)
Education	
Illiterate	105 (26.7)
Read and write	49 (12.5)
Primary school complete	142 (36.1)
≥ Secondary school complete	97 (24.7)
Livelihood	
Civil servant	49 (12.5)
Farmer (private)	171 (43.5)
Merchant	48 (12.2)
Student	66 (16.8)
Private employee	6 (1.5)
Housewife	42 (10.7)
Daily laborer	11 (2.8)
House roof material	
Thatch/straw	104 (26.5)
Iron sheets	289 (73.5)
House wall material	
Wood with mud	381 (97.0)
Wood only	8 (2.5)
Wood with mud and cement	4 (1.0)

While most of the questions focus on knowledge, a few refer to actual malaria prevention and treatment practices (e.g. treatment seeking health facility, malaria control tools). The majority (86 %) of the respondents cited that mosquitoes bite humans at night during bedtime. A similar figure (89.8 %) replied that malaria is a preventable disease. Two-hundred and seventy-six (70.2 %) of the respondents reported that September–November is the notable malaria transmission season; 345 (87.8 %) of the interviewees mentioned stagnant water as a principal mosquito breeding site. Among the respondents, 99.2 % (390) perceived that malaria is fatal if not treated. Most (88.8 %) of the respondents practiced use of mosquito nets, followed by draining of collected water bodies (79.6 %), house spray with insecticide (73.0 %) and closing doors and windows in the evening (42.2 %) to prevent malaria. All respondents had heard about malaria although sources could vary. Health workers were sources of knowledge cited by most (74.0 %) of the respondents, followed by radio (41.9 %), school (28.8 %), friends (24.7 %), national television (20.6 %), government newspaper (10.9 %), and parents (9.2 %). Children below the age of five years were perceived to be most susceptible to malaria by 81.2 % of the interviewees, followed by pregnant women (cited by 54.2 %), all humans with any age category (33.3 %), all adults (31.3 %) and old ages (26.2 %). The youths were identified as the most susceptible to malaria group in communities by 24.2 % of the interviewees. About 78.8 % of the respondents knew that mosquito bites are responsible for malaria transmission. However, 18.1 and 3.1 % of the respondents associated the cause of malaria with exposure to rain and body contact with other malaria patients, respectively.

All respondents knew the kind of anti-malarial drug used for treatment. Specifically, 79.6, 19.6 and 0.8 % of the respondents mentioned coartem, chloroquine and fansider as anti-malaria drugs of choice, respectively (Table 4). All respondents reported the practice of IRS in their houses prior to the 2013 malaria transmission season. Among these, 49.4, 48.1 and 2.5 % reported a practice of IRS in their houses within 3, 6 and 12 months, respectively (Table 5). Of the 393 HHs 388 (98.7 %) owned at least one bed net. Among the owners, 265 (68.3 %), 110 (28.4 %) and 13 (3.3 %) had one, two and three LLINs per HH, respectively, with the mean number of LLINs owned being 1.3. Among the LLIN owners, 72.5 % believed that LLINs can avoid mosquito bites. Of the HHs that owned LLINs, 72.9, 48.7, 41.5, 36.3, and 13.6 % were used (the previous night) by the under-five children, pregnant women, old ages, youths and 5 to14 years old, respectively.

**Table 4:** Malaria related knowledge, attitude and practice of inhabitants interviewed (*n* = 393) in Walga Health Center catchment area, Abeshge District, South-Central Ethiopia, 2013

Variables	Frequency (%)
Untreated malaria is fatal (knowledge)	
Yes	390 (99.2)
No	3 (0.80)
Malaria is preventable (knowledge)	
Yes	353 (89.8)
No	40 (10.2)
Malaria control tools (knowledge as well as actual practice)	
ITN	349 (88.8)
IRS	10 (2.5)
Drug	287 (73.0)
Close widow/door at night	166 (42.2)
Use traditional medicine	9 (2.3)
Keep HH hygiene	3 (0.8)
Drain stagnant water	313 (79.6)
Mosquito biting time (knowledge)	
Daytime	9 (2.3)
Nighttime	338 (86.0)
Both day and nighttime	8 (2.0)
Do not know	38 (9.7)
Mosquito breeding site (knowledge)	
Stagnant water	345 (87.8)
Running water	37 (9.4)
Bushes	11 (2.8)
Seek treatment from (actual practice)	
Health centre	126 (76.4)
Health post	26 (15.7)
Hospital	13 (7.9)
Malaria symptoms (knowledge)	
Fever	365 (92.9)
Shivering	193 (49.1)
Chills	218 (55.4)
Headache	294 (74.8)
Tiredness	92 (23.4)
Loss of appetite	113 (28.8)
Joint pain	127 (32.3)
Know malaria drugs (knowledge)	
Yes	393 (100)
No	0 (0.0)
Malaria drugs (knowledge, practice)	
Coartem	313 (79.6)
Chloroquine	77 (19.6)
Fansidar	3 (0.8)

**Table 5:** ITN and IRS related information in the four selected Walga Health Centre catchment area Abeshge District, South-Central Ethiopia during the study period, 2013

Description	Frequency (%)
ITN possessed	
Yes	388 (98.7)
No	5 (1.3)
ITN used last night	
Yes	388 (100)
No	0 (0.0)
ITNs per family	
One	265 (68.3)
Two	110 (28.4)
More than two	13 (3.3.)
Washed ITN in HH	
Yes	388 (98.7)
No	5 (1.3)
Washings	
Monthly	94 (24.2)
Every 3 months	107 (27.6)
Every 6 months	187 (48.2)
Why frequent washing	
Net gets easily dirty	345 (88.9)
Removes bad smell	43 (11.1)
IRS	
Last 3 months	194 (49.4)
Last 6 months	189 (48.1)
Last 12 months	10 (2.5)
Never	0 (0.0)
Benefits of ITN	
Stops mosquito bite	309 (78.6)
Prevents malaria	84 (21.4)

## Discussion

The significant rise in malaria prevalence in 2009 compared to 2008 in the study area may suggest a history of some low-level epidemics attributable to differences in climatic, environmental or human behavioural risk factors in the 2 years. Registering significantly higher number of febrile patients in 2010, in the occurrence of significantly lower malaria prevalence, in contrast to both 2009 and 2008 suggests the rise of non-malaria-related febrile illnesses in 2010 in the locality. In general, despite a fluctuating trend, the health system data showed a successive and significant decline in malaria prevalence starting from 2009. This significantly declining pattern of malaria cases in the studied healthcare setting coupled with the low prevalence rate observed among the study participants in the current community-based cross-sectional study, demonstrates the effectiveness of the control measures being implemented in the area. Integrated control efforts are underway in the district as part of the nationwide anti-malaria campaign. From 2011 onwards, the reduction of malaria cases coincides with the increased availability of the new, effective, anti-malarial drug, artemisinin combination therapy (ACT), the increased political commitment and community awareness concerning malaria control intervention. Scale-up of ACT as a first-line anti-malarial drug combined with vector control using IRS, ITNs/LLINs, draining of stagnant water and good community KAP resulted in a dramatic decline in the malaria burden in Eritrea [35], Kenya [36] and from other endemic parts of Ethiopia [37].

The lower malaria prevalence (0.25 %) observed in this study compared to prevalence reports from Butajira area (0.93 %), another endemic focus in Gurage Zone [38], Shewa Robit in northeast Ethiopia (2.8 %) [39], from various other parts of Ethiopia in malaria indicator survey (1.0 %) [17], and Kenya [36] (18.0 %) might be due to environmental variations and type of study populations. Differences in the scope of these studies, climatological differences, altitudinal variations, variations in the methods of malaria diagnosis, differences in the skills and overall competence of the microscopists, differences in the coverage and utilization of intervention tools, community awareness (or KAP), human/parasite genetics, presence of co-infection and other factors, may explain the apparent malaria prevalence differences reported by various investigators. For instance, in this study, participants consisted of all age groups, but those of Peter et al. [36] in Kenya were pregnant women who are at-risk groups.

In general, this study has generated evidence in support of the effectiveness of the ongoing control interventions in the area. The 0.25 % malaria prevalence in this HH survey showed a 2.5 % reduction compared with the average yearly prevalence (33.8 %) and monthly estimate (2.8 %) from the health centre’s record, demonstrating further shrinking of malaria in the area, notwithstanding sub-microscopic infections and the fact that the study was conducted in the dry season and it was community-based. However, since about 82 % of the districts’ total landmass is malarious the risk of malaria is high. This is in line with the current national malaria picture where the disease is still a leading cause of outpatient consultations while a dramatic reduction in overall malaria burden is noticed across Ethiopia [2].

The retrospective data of the present study revealed that significantly more male febrile patients attended the health centre with more malaria prevalence, although not significant, suggesting sex-related occupational and behavioural differences as well as travel history. Although prevalence data for each age group is missing, 44.9 % of the malaria patients were over 14 years and those 5 to 14 years old were the least affected. This might be because of increased visits to the health centre by the older individuals, differences in treatment-seeking behaviour, occupational and behavioural risk factors, such as staying outdoors before bedtime for various reasons. The most productive segment of the community, individuals older than 14 years of age, and more males might be more affected due to the fact that these two groups often stay outdoors at night for work and go to bed late in the night. People who work in the field during the night have little practice of personal protective measures, such as repellents and protective clothing. As people get older their sleeping patterns may be less regular and they may therefore be more at risk of infective mosquito bites. Moreover, some older individuals might have been more vulnerable as a result of lower immunity.

From the retrospective data, although there was some evidence of seasonality in malaria transmission, the considerable number of cases in the dry season implies the existence of favourable local conditions or hotspots for perennial transmission. The presence of year-round mosquito-sustaining micro-environments or hotspots in the dry season must be investigated. Travel history of the residents to nearby or other perennial transmission settings might account for the almost year-round visit of patients to WHC. The continuous presence of *P. vivax* might be explained by its ability to relapse. Also, a possible recrudescence cannot be ruled out for *P. falciparum* in both dry and wet seasons*.*

Although both species were commonly encountered in the study area, the overall significant variation demonstrates the relative dominance of *P. falciparum* over *P. vivax*. But it is difficult to explain the scarcity of mixed infections of the two species. Most of the cases tended to be mono-infections. Further, there was distinct and opposing seasonal variation in the relative dominance of the two parasites. The season where the highest *P. falciparum* cases were reported was the lowest for *P. vivax* and vice versa. It is difficult, for instance, to explain why *P. vivax* cases were lowest between March and May, which is the ‘small rains’ season, and *P. falciparum* was expectedly more frequently detected. Taken together, the paucity of mixed infections and opposing seasonal occurrences may suggest a probable antagonistic or competitive/inhibitory effect of one species on the other. Possible differences in temperature-dependent developmental and reproductive biology of the parasites in the mosquito vector, as well as man, may also explain such findings. In 2008 and 2011, *P. vivax* was significantly higher in the study area and the opposite was true for 2010 and 2012. In 2009 the two species had frequency comparable. The predominance of *P. vivax* might be due to its relapse or drug efficacy against *P. falciparum.* Further, some diagnostic problems associated with *P. falciparum* because of such behaviour as rossetting may result in false-negative results. The findings demonstrate the increased public health importance of vivax malaria in the locality. The upsurge of *P. falciparum* in 2010 and 2012 compared to *P. vivax* might be due to some treatment failures and/or recrudescence.

The questionnaire survey results showed that all respondents had heard of malaria and 99.2 % of them believed that malaria was one of the most important health problems of the community affecting both sex and all age groups. This is consistent with previous reports from other parts of Ethiopia [39]. Most of the respondents were also familiar with at least one of the established symptoms of malaria, which is commonly expected of populations in endemic areas. The present finding also supported studies from Butajira [38] and Shewa Robit [39], who reported that fever, headache, shivering and chills were understood by the respondents as signs and symptoms of uncomplicated malaria. The majority of the respondents (78.9 %) mentioned that mosquito bites were responsible for malaria transmission. This awareness is higher than the KAP study conducted in various parts of Ethiopia, such as Assosa [40] and Gondar [41], 48 and 74 %, respectively, but it is lower than that in Shewa Robit (85.5 %) [39] and northern Swaziland, 99.7 % [42]. The awareness of the respondents that malaria is transmitted by the bite of a mosquito is usually common knowledge in malaria-endemic countries, such as India [24], Ghana [43] and Sudan [44]. The difference might be due to differences in the study sites and some respondent characteristics. In Abeshge, the respondents were rural people and urban dwellers have expectedly better access to information, education and communication or have behavioural adaptability to control the disease.

In Ethiopia, the regular practice of creating community awareness about health issues through health extension workers was launched recently. Although a much higher number of respondents (78.9 %) had mentioned mosquito bite as the cause of malaria, 21.1 % had replied that exposure to rain and body contact with malaria patients could cause malaria, showing that the understanding of the cause of malaria could vary from community to community and among individual HHs. This suggests that misconceptions concerning malaria prevail and that practices in the control of the disease are inadequate, which may affect malaria control effectiveness and sustainability. It is recommended that health education interventions should be designed according to the existing knowledge and awareness level, as well as treatment-seeking practices, of at-risk populations, and should be implemented for a sufficient length of time to be effective [45].

Eighty-six percent of the participants in the present study mentioned mosquitoes’ habit of biting during sleeping time and 87.8 % of the respondents knew stagnant water bodies as breeding sites for mosquitoes. This result is higher than was observed in Butajira, central Ethiopia, [38] and northern Ethiopia [46] where 79.8 and 72.6 % of the study participants, respectively, cited that mosquitoes are mainly believed to breed in stagnant water. In Sudan only 73.2 % of respondents knew that mosquitoes bite human beings at nighttime [47]. The increased correct perception among respondents of the present study to take appropriate preventive measures and proper use of mosquito nets is encouraging. Regarding treatment seeking, all respondents mentioned that health facilities were the most common sources for treatment. This is consistent with other observations in other African countries [48] and India [24] where health facility services were preferred most frequently when malaria is suspected [48].

Regarding malaria prevention options, 89.8 % of the study participants were of the view that malaria is preventable. This coincides with a study in Swaziland [49], Nepal [50] and in north Ethiopia [46] where 78, 86.4 and 90.8 % of the respondents, respectively, had a similar view. In terms of drugs, 79.6, 19.6 and 0.8 % of the study participants were likely to know coartem, chloroquine and fansider as anti-malarial drugs, respectively. The study showed that 100 % of the study participants agreed with the statement “the best way to treat malaria is to take appropriate drugs”. Use of mosquito nets (88.8 %), draining stagnant water (79.6 %) and IRS (73.9 %) were the three main types of malaria preventive measures frequently reported by the current study participants.

The observation that 98.7 % of the presently surveyed HHs possessed ITNs is in line with the national coverage [2] and among these, 285 (72.5 %) understood the purpose properly and hence made use of it to prevent malaria. While current result was nearly consistent with a report from Eritrea [51] concerning the proportion of pregnant women sleeping under ITN, it was higher for the under-five children. These same authors [51] revealed that ITN use as a single intervention tool achieved 84 % decline in malaria morbidity and mortality.

All of the respondents reported that they had washed their INTs at least three times in a year. The findings of a study in southern Ethiopia indicated that frequent washing reduced ITN effectiveness by about three-fold [52]. Review of randomized trials also revealed similar findings in that frequent washing reduced the concentration of permethrin on the netting materials and the killing and repelling abilities of the insecticide and thereby reduced the effectiveness of ITNs [15]. Therefore, the community needs to be educated about the counterproductive effect of washing their nets *vis-à-vis* protection from malaria. However, all respondents knew that IRS prevents malaria and that they get their houses sprayed every six months following both the major and minor rainy seasons. This indicates that the acceptance rate for IRS is higher compared with findings from a study from north Ethiopia, which was 42.7 % [46].

Taken together, the high intervention coverage coupled with increased community awareness and participation resulted in low malaria prevalence in the study area. Nonetheless, the cross-sectional design of the study with a focus on the dry season and afebrile individuals might have accounted for the lower yield in the parasitological survey. Also, the slide results, which may have missed submicroscopic infections, were not confirmed by polymerase chain reaction. Furthermore, 5-year data may not be robust enough to generalize on long-term trends. The initial plan was to analyse a 10-year trend of malaria prevalence in the study area, but this was not possible due to poor recordkeeping and reporting system. These could be considered as limitations of the study.

## Conclusions

Between 2009 and 2012, malaria prevalence showed a significant decline in the study area and the community-based active case detection survey identified a single slide-positive case. Apart from the very high LLIN coverage, insecticide spraying and malaria case detection and treatment, KAP results revealed that the community had high awareness about the transmission, symptoms and prevention of malaria. Knowledge-based environmental management is also underway in the study area. The data show the magnitude of malaria control tools and their effectiveness in the area; and if this achievement could be sustained, planning for pre-elimination of the disease in the locality may be feasible in the near future.

## Abbreviations

WHC: Walga Health Centre
KAP: Knowledge, attitude and practice
masl: Meters above sea level
ITNs: Insecticide-treated bed nets
LLINs: Long-lasting insecticidal nets
IRS: Indoor residual spraying
HHs: Households
ACT: Artemisinin-based combination therapy
